# SERS Properties of Different Sized and Shaped Gold Nanoparticles Biosynthesized under Different Environmental Conditions by *Neurospora crassa* Extract

**DOI:** 10.1371/journal.pone.0077486

**Published:** 2013-10-09

**Authors:** Katrin Quester, Miguel Avalos-Borja, Alfredo Rafael Vilchis-Nestor, Marco Antonio Camacho-López, Ernestina Castro-Longoria

**Affiliations:** 1 Departamento de Microbiología, Centro de Investigación Científica y de Educación Superior de Ensenada (CICESE), Ensenada, B.C., Mexico; 2 Centro de Nanociencias y Nanotecnologia (CNyN), Universidad Nacional Autónoma de México (UNAM), Ensenada, Mexico; 3 Centro Conjunto de Investigación en Química Sustentable (CCIQS), UAEM-UNAM, Toluca, Mexico; 4 Laboratorio de Investigación y Desarollo de Materiales Avanzados, Sección de Espectroscopía, Raman, Facultad de Química, UAEMex, Toluca, Estado de México, Mexico.; Massey University, New Zealand

## Abstract

Surface-enhanced Raman scattering (SERS) is a surface-sensitive technique that enhances Raman scattering by molecules adsorbed on rough metal surfaces. It is known that metal nanoparticles, especially gold and silver nanoparticles, exhibit great SERS properties, which make them very attractive for the development of biosensors and biocatalysts. On the other hand, the development of ecofriendly methods for the synthesis of metallic nanostructures has become the focus of research in several countries, and many microorganisms and plants have already been used to biosynthesize metallic nanostructures. However, the majority of these are pathogenic to plants or humans. Here, we report gold nanoparticles with good SERS properties, biosynthesized by *Neurospora crassa* extract under different environmental conditions, increasing Raman signals up to 40 times using methylene blue as a target molecule. Incubation of tetrachloroauric acid solution with the fungal extract at 60°C and a pH value of a) 3, b) 5.5, and c) 10 resulted in the formation of gold nanoparticles of a) different shapes like triangles, hexagons, pentagons etc. in a broad size range of about 10-200 nm, b) mostly quasi-spheres with some different shapes in a main size range of 6-23 nm, and c) only quasi-spheres of 3-12 nm. Analyses included TEM, HRTEM, and EDS in order to corroborate the shape and the elemental character of the gold nanoparticles, respectively. The results presented here show that these ‘green’ synthesized gold nanoparticles might have potential applicability in the field of biological sensing.

## Introduction

Surface-enhanced Raman scattering (SERS) is an effective technique for the study of surface/interfacial properties, and the interaction between biomolecules and metal surfaces has recently given rise to a large amount of research [1]. For example, it was shown that gold nanoparticles (NPs) showed great SERS properties in the detection of oral cancer cells [2], and silver nanoprisms and gold NPs showed excellent results in DNA detection via SERS [3]; however, the NPs used were synthesized by chemical reduction, which, including other chemical and physical methods for NP formation, is regarded as having a relatively high environmental cost since these methods are often energy intensive, employ toxic chemicals, and require higher temperature [4,5]. Therefore, it is important to develop clean and environmentally benign processes for nanomaterial synthesis. This so-called ‘green chemistry’ or nanobiotechnology employs biological systems like microorganisms to fabricate nanostructures and has the additional benefit of improving the biocompatibility of nanomaterial [6]. Nanostructure biosynthesis assisted by biological systems follows three principles: the organism is (i) eco-friendly as are (ii) the reducing agent employed and (iii) the capping agent in the reaction [7].

Nanostructures have several important applications in various fields, including sensor technology [8], optical devices [9], catalysis [10], biological labeling [11], and drug delivery systems [12]. The magnetic, electronic, and optical properties of metallic nanostructures are closely related to their size, shape, size-distribution, and surrounding environment. Therefore, for a biological process to successfully compete with chemical and physical nanostructure synthesis, very strict control over average particle size in a specific size range and uniform particle morphology is required but still remains a challenge [13]. Many publications report the successful synthesis of metal nanostructures by a biological system [14-18]; however, to date only very little work has been conducted on the actual mechanisms and manipulation of nanostructure formation in microorganisms [19]. So far, few studies have been conducted on the application of biosynthesized nanoparticles in SERS. One of the first publications reporting successful Raman enhancement by triangular gold NPs, synthesized by pelargonium plant extract, with p-aminothiophenol as probe molecule was reported in 2008 [20], and spherical gold NPs, synthesized by isolated chloroplasts, were reported to successfully enhance Raman signals of Rhodamine 6G [21]. Also intracellular SERS by gold NPs synthesized in the bacterium *Geobacter sulfurreducens* [22] and the fungus *Aspergillus nidulans* [23] was performed showing the potential of this method to be used in targeted analysis of intracellular compartment analysis in the future.

Previous work demonstrates that the non-pathogenic filamentous fungus *Neurospora crassa* is a good candidate to further explore a finer protocol for the synthesis of metallic nanostructures [24,25]. Therefore, in this work, we present the use of *N. crassa* extract to successfully biosynthesize gold nanostructures under different environmental conditions such as temperature, pH value, and incubation time, as well as combinations of all the mentioned conditions. Analyses to confirm the elemental character of gold NPs include high-resolution transmission electron microscopy (HRTEM) and energy dispersive X-ray spectroscopy (EDS). Gold NPs were produced to explore their possible use in surface-enhanced Raman scattering (SERS). Results clearly show that they exhibit good SERS properties, increasing Raman signals up to 40 times using the dye methylene blue (MB) as a target molecule.

## Materials and Methods

### Preparation of fungal extract

Macroconidia of *N. crassa* (FGSC # 9013) were inoculated in 100 mL potato dextrose broth (PDB, obtained from Difco) and incubated at 28°C for 4 days under gentle agitation in an orbital shaker (Orbit Environ Shaker) at 125 rpm. Grown biomass was filtered and washed 3 times each in sterile distilled and deionized water. The biomass was ground 1:1 in deionized water (w/v), centrifuged for 5 minutes at 6500 rpm, filtered through a 0.2µm microfilter, and stored at 4°C until use (not more than 7 days). Fungal extract protein content was quantified by the Bradford Assay [26] in triplicate.

### Synthesis of gold nanoparticles

10^-3^M HAuCl_4_ (Sigma-Aldrich) aqueous solution was added to the fungal extract with a protein content of 1.7 mg/mL at a ratio of 1:3 (fungal extract: metal precursor) and incubated under different pH values (3, 5.5, and 10) and temperatures (4, 25, 37, 60, and 80°C), as well as all possible combinations, in the dark for different time periods (3, 6, 9, and 12 hours). The pH value was modified by adding 1 M HCl or NaOH aqueous solution.

### TEM analysis

The synthesized gold nanostructures were analyzed under Hitachi H-7500 and JEOL 2010 transmission electron microscopes. HRTEM, HAADF, and EDS analyses were performed in a FEI Tecnai F30 electron microscope. Particle size diameters were calculated with the equation *d*_*avg*_= *∑(n*_*i*_ *d*_*i*_)/*∑n*_*i*_, where n_i_ is the number of particles with a diameter d_i_. 100 NPs were determined each. The atomic distance was determined using the software Gatan DigitalMicrograph^TM^.

### Statistical analysis

An analysis of variance (ANOVA) of the data obtained was performed by using the software Statistica 7. Significance was calculated by Tukey’s HSD (Honestly Significant Difference) *post hoc* test. Confidence intervals were set to 0.95.

### SERS

The resulting extract-NP solution was dried overnight at room temperature on a glass microscope slide. As negative control, fungal extract only was used. All samples were burned at 600°C for 1 hour with previous intervals of 20 min each at 100, 200, 300, 400, and 500°C to eliminate the organic fraction of the samples and thereby purify the NPs. 25 mg of methylene blue was prepared in 200 mL methanol. The glass slides carrying the gold NPs were incubated in the methylene blue solution overnight and then dried for 5 min on a heater at 100°C. Raman spectra were collected with a microRaman system (LabRam HR-800 of Jobin Yvon Horiba). A He-Ne laser (λ=632.8 nm) was used to excite the sample with an intensity of 1.5 mW. A CCD camera was used as a detector. Data acquisition time was 60 s with 10 accumulations. The same experimental parameters were used for all samples.

## Results and Discussion

**Figure 1:**
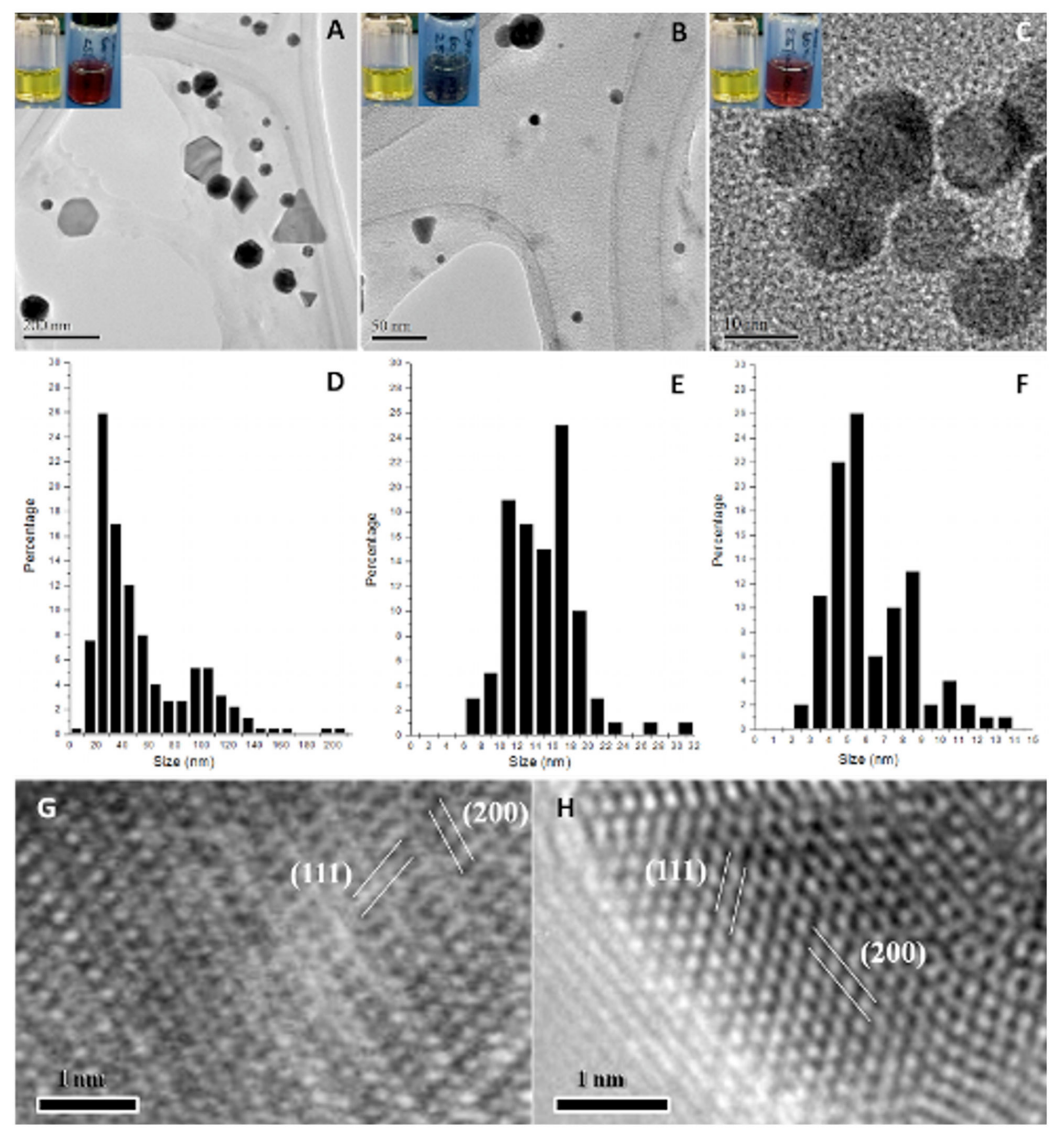
TEM analysis. Gold nanoparticles synthesized by *N. crassa* extract at 60°C and pH 3 (A), pH 5.5 (B), and pH 10 (C). Insets: color change from before (yellow; left image) to after (red / purple; right image) incubation. D-F: Corresponding size distribution histograms of formed nanoparticles. G-H: HRTEM images of a quasi-spherical (G) and a hexagonal (H) gold nanoparticle with plane information. Gold nanoparticles synthesized by *N. crassa* extract at 60°C and pH 3 resulted in the formation of different shapes in a broad size range, incubation at 60°C and pH 5.5 results in the formation of mostly quasi-spherical particles and some different shapes, mainly triangles, with a main size range of 6 to 23 nm, and incubation at 60°C and pH 10 shows formation of quasi-spherical particles in a very small size range (3 to 12 nm).

The development and understanding of ecofriendly methods to synthesize metal nanoparticles are the subject of current research in various countries. Thus, in this work, we present the employment of *N. crassa* extract for the biosynthesis of gold NPs of different size and / or shape in order to test their applicability in SERS. Incubation of the fungal extract with tetrachloroauric acid solution using different temperatures and pH values as well as all possible combinations of these resulted in the formation of different-sized and -shaped NPs (Figure 1). Under all conditions applied, it came to nanostructure formation, but, since our interest lies in discovering protocols how to manipulate size and shape, only promising results showing a small-sized or different-shaped NP formation are reported here. After 9 hours, there was no apparent change in the amount of NPs compared to longer incubation times. Therefore, the results presented here were obtained using an incubation time of 9h, temperature of 60°C and the pH values a) 3 (Figure 1A and D), b) non-modified (5.5; Figure 1B and E), and c) 10 (Figure 1C and F). Figure S1 shows results of statistical analysis (ANOVA) of gold NPs obtained at 60°C under the applied pH values (3, 5.5, and 10), revealing that although NPs were already formed after 3 hours, differences of quantity but no significant differences in size and / or shape of the formed NPs were detected (panel A). Significant differences according to Tukey’s HSD *post hoc* test were calculated with confidence intervals of 0.95 and marked in red (panel B) (Figure S1). Clearly, NPs obtained at 60°C and pH 3, are significantly bigger than those obtained at 60°C and pH 5.5 or pH 10, respectively, while no significant differences were observed between pH 5.5 and 10.

Additionally, gold NP stability was analyzed over time. TEM images show that they kept their size and shape for over 10 months stored at room temperature (Figure S2 shows size distribution histograms of gold NPs synthesized at 60°C and pH 3 (A), pH 5.5 (B), and pH 10 (C) after more than 10 months at room temperature; Figure S2).

### HRTEM analysis of gold nanoparticles

**Figure 2:**
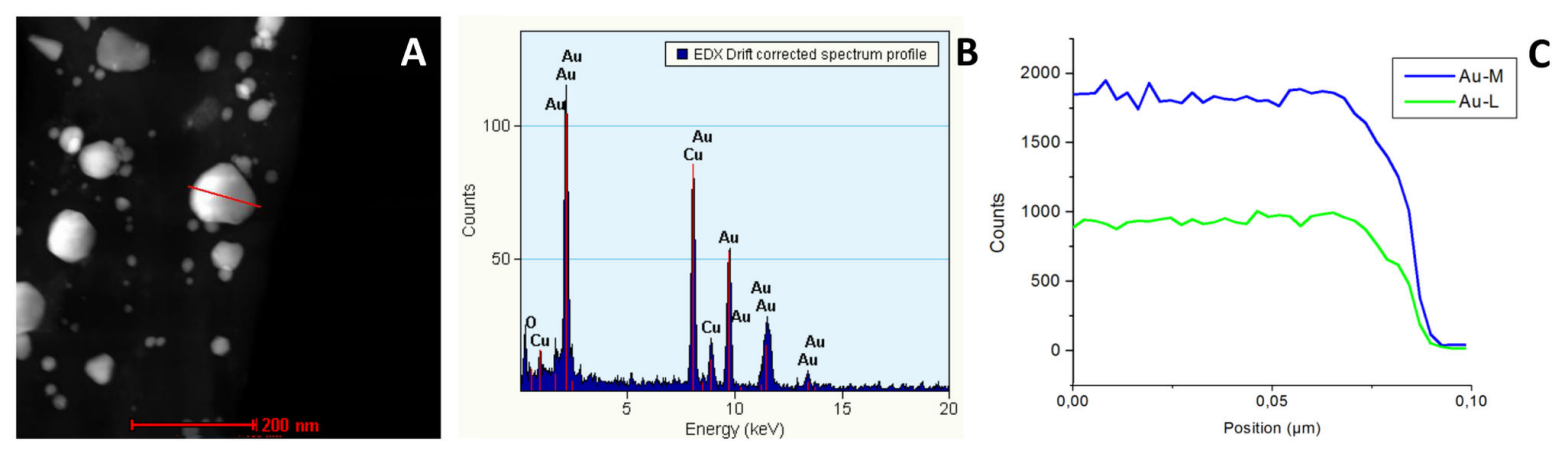
EDS spectrum. Representative EDS spectrum of synthesized gold nanoparticles. A: HADDF image of gold nanoparticles. B and C: lineal EDS spectrum.

Successful biosynthesis of gold nanostructures was already indicated by the color change of the solution from yellow to red / purple upon exposure to the HAuCl_4_ precursor solution (insets of Fig. 1A to C). Gold NP synthesis at 60°C and a pH of 3 resulted in the formation of different shapes such as spheres, triangles, hexagons, pentagons, etc. (Fig. 1A) in a broad size range of about 10 to 200 nm edge length (Fig. 1D). Figure 1B shows the formation of quasi-spherical gold NPs with a main size range of about 6 to 23 nm (Fig. 1E) using a synthesis temperature of 60°C without modifying the pH value, which was 5.5. Also, the formation of some different-shaped NPs, mainly triangles, was noticed but the majority showed quasi-spherical character. Using a pH value of 10 at 60°C resulted in the formation of only quasi-spherical gold NPs (Figure 1C) in a very small main size range of 3 to 12 nm (Figure 1F). Determination of lattice fringe spacing of a quasi-spherical (Figure 1G) and a hexagonal gold NP (Figure 1H) confirms the planes (111) and (200) of face-centered cubic (fcc) metal gold while EDS analysis (Figure 2) confirmed the elemental character of synthesized gold NPs.

Results indicate that the formation of different sizes and / or shapes can be tuned by combining a range of temperatures with varying pH value. It was already shown that by varying the pH value and / or temperature, the resulting shapes and / or sizes of metal NPs synthesized by fungi vary [6,27-29]; however, the fungal strains used are potential plant or human pathogens. In this work, we use the extract of the non-pathogenic fungus *N. crassa* and we show that an alkaline pH value at a temperature of 60°C favors the formation of small (3 to 12 nm), quasi-spherical gold NPs while acid pH value resulted in the formation of different-shaped NPs in a broad size range of about 10 to 200 nm. Using a pH value which was not modified (5.5) resulted in the formation of quasi-spherical nanostructures of 6 to 23 nm with some different shapes, mostly triangles. The fact that high pH values favored the formation of quasi-spherical gold NPs was also reported by the use of cell-free extract of *Aspergillus oryzae* var. *viridis* [30], although the reaction temperature was 25°C. In the same experiment, it was shown that acidic pH did not favor the synthesis of Au NPs, which on the other hand did not coincide with our results.

In general, proteins are assumed to be responsible for the bioreduction and for stabilizing and capping the newly formed nanostructures [6,31,32]. Bioreduction results in the formation of Au cores, which then grow to NPs while being stabilized by proteins. As mentioned before, we use fungal extract for NP synthesis, which is a solution containing a high proportion of soluble fungal proteins (1.7 mg protein / mL *N. crassa* extract, as shown by the Bradford assay [26]). The exact reduction mechanism remains unknown, but at a high temperature of 60°C, it is probable that all proteins are denatured and thereby lose their 3-dimensional structures, which indicates that all functional groups necessary for bioreduction and stabilization are accessible.

Deprotonated carboxyl groups are proposed to be able to stabilize metal NPs due to their outside electron conformation [33]. At low pH values, the carboxyl groups are protonated and neutrally charged [29], which might decrease their stabilization capacity, thereby favoring anisotropic NP growth, resulting in the formation of bigger and different shaped NPs such as triangles, hexagons, and pentagons (Figure 1A). However, at high pH values, an equal stabilization might be achieved due to deprotonated carboxyl groups, favoring isotropic growth of gold NPs due to increased stabilization capacity, resulting in the formation of small, quasi-spherical particles (Figure 1C). At unmodified pH (5.5, Figure 1B), it comes to the formation of quasi-spherical as well as few different shaped gold NPs, indicating that most carboxyl groups are deprotonated, thereby resulting in isotropic growth of quasi-spherical shapes, with the occurrence of some protonated carboxyl groups, being responsible for the formation of a few different-shaped NPs due to anisotropic growth.

### SERS

**Figure 3:**
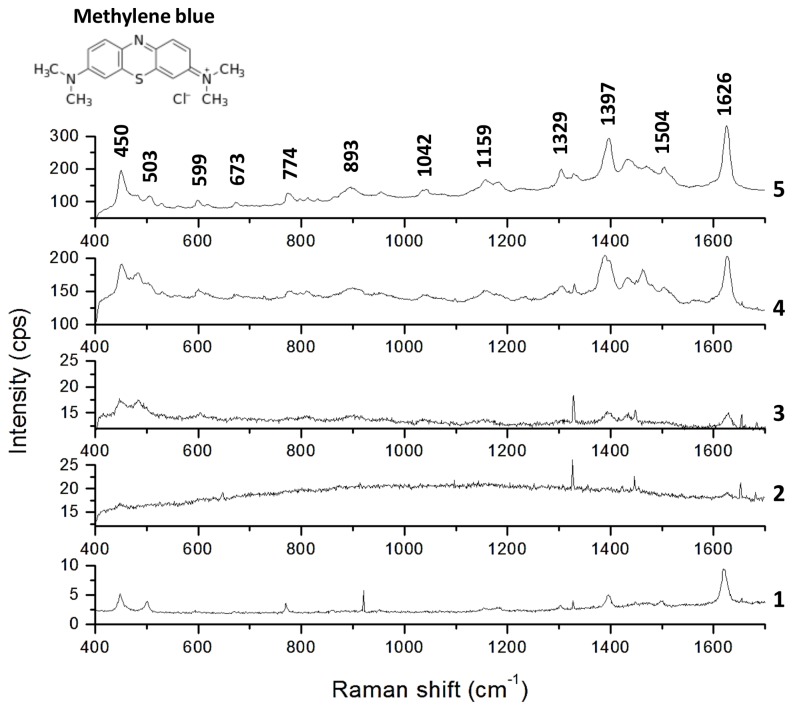
SERS. Raman spectra of MB (1), MB + fungal extract (2), MB + quasi-spherical AuNPs of 3 to 12 nm (3), MB + quasi-spherical AuNPs with some different shapes of 6 to 23 nm (4), and MB + different shaped gold nanoparticles with a broad size range (5).

We evaluated the performance of the three above-mentioned types of gold NPs synthesized by *N. crassa* extract for SERS application using the dye methylene blue as a target molecule. Before analysis, gold NPs were purified by heat-elimination of the organic content. Figure 3 shows the Raman spectra of methylene blue on the gold NPs synthesized in this work compared to the spectra of methylene blue only and a control. Shown are spectra of MB (spectrum 1), MB on control (fungal extract only, spectrum 2) and MB on the three different gold NP samples (spectra 3 to 5). The synthesized gold NPs of different shapes are shown to possess much stronger SERS enhancement capacity relative to quasi-spherical gold NPs. Quasi-spherical NPs of 3 to 12 nm, synthesized at 60°C and pH 10, enhance Raman signals of methylene blue about 2 times (spectrum 3), while mostly quasi-spherical NPs with some different shapes in a main size range of 6 to 23 nm, synthesized at 60°C and pH 5.5, enhance Raman signals about 25 times (spectrum 4). NPs of different shapes, like triangles, hexagons and pentagons, in a broad size range of approximately 10 to 200 nm, synthesized at 60°C and pH 3, enhance Raman signals of methylene blue about 40 times (spectrum 5). Since the control spectrum (MB on fungal extract without gold NPs; spectrum 2) is similar to the one recorded with methylene blue only (spectrum 1), we can conclude that the gold NPs contribute to the SERS effect observed and not some remaining organic content of the extract solution. To exclude the formation of any agglomeration due to the heat treatment, we recollected Au NPs after SERS measurements by adding deionized water to the glass slides and transferring them to carbon-coated copper grids. TEM analyses showed that up to 90% of the Au NPs kept their size and shape after heat treatment (data not shown). Low percentages showed bigger sizes, which might be explained by the stronger reactivity of Au NPs due to the elimination of the stabilizing agent by the heat treatment, since it is known that proteins used as stabilizing agents prevent agglomeration [34]. Furthermore, it is supposed that if the heat treatment resulted in NP aggregation, the Raman spectra of all samples would show a similar enhancement, which also is not the case.

The peak at 451 cm^-1^ with a shoulder peak at 504 cm^-1^ corresponds to skeletal deformation vibrations of C-N-C and C-S-C [35-37]. C-H out-of-plane bending is responsible for the peak at 683 cm^-1^ [36,37], and the peak at 774 cm^-1^ appears due to C-N-C and C-S-C skeletal deformation as well as N-CH_3_ stretching [37]. The peak appearing at 1011 cm^-1^ corresponds to C-H in-plane bending [35-37] and the Raman signal at 1160 cm^-1^ might appear due to C-H out-of plane bending [35] or C-N stretching [37]. The peak at 1335 cm^-1^ corresponds to C-H in-plane ring deformation [36], and we detected a Raman signal at 1402 cm^-1^ corresponding to asymmetric C-N stretching [39]. The peaks at 1505 cm^-1^ and 1626 cm^-1^ correspond to C-C-C asymmetric skeletal deformation [39] and C-C ring stretching [36,39], respectively. Furthermore, in the spectra of MB on gold NPs, we detected additional peaks that were not observed in the MB spectra. The one at about 600 cm^-1^ corresponds to C-N-C skeletal deformation [36,37] while the corresponding vibrational mode of the peak at about 900 cm^-1^ could not be found in the literature. This peak might result from a displacement of the Raman signal at 774 cm^-1^ or 1011 cm^-1^. Another possible explanation is that this peak appears due to small amounts of remaining biomass, which would imply that not all of the organic fraction could be eliminated by the heat treatment; however, this possibly remaining fraction is regarded as insignificantly low. An overview of the vibrational modes of MB corresponding to its Raman spectrum is shown in Table 1.

**Table 1:** Vibrational modes of MB corresponding to its Raman spectrum.

**MB (cm^-1^)**	**MB + AuNPs-1(cm^-1^)**	**MB + AuNPs-2(cm^-1^)**	**MB + AuNPs-3(cm^-1^)**	**Reported results (cm^-1^)**	**Peak assignment**
451	450	447	447	480^a^ 445^b^ 449^c^	δ(C-N-C), δ(C-S-C)
504	503	482	483	497^b^502^c^	δ(C-N-C)
-	599	597	605	613^a^	δ(C-N-C)
683	673	671	675	677^b^ 688^c^	γ(C-H)
774	774	774	773	769^c^	δ(C-N-C), δ(C-S-C), ν(N-CH_3_)
-	893	895	902	-	-
1011	1042	1039	1034	1032^a^ 1036^b^ 1003^c^	β(C-H)
1160	1159	1154	1154	1123^a^ 1181^b^	γ(C-H) ν(C-N)
1335	1329	1299	-	1396^b^	α(C-H)
1402	1397	1386	1391	1413^d^	ν_asym_(C-N)
1505	1504	1501	-	1512^e^	δ_asym_ (C-C-C)
1626	1626	1623	1626	1618^b^ 1617^d^	ν (C-C) ring

Shown are Raman shifts (in cm^-1^) of methylene blue on the different gold NP samples compared to reported data. AuNPs-1: Different shaped nanoparticles of a broad size range (10 to 200 nm). AuNPs-2: Quasi-spherical nanoparticles with some triangular shapes in a main size range of 6 to 23 nm. AuNPs-3: Small, quasi-spherical nanoparticles (3 to 12 nm). Abbreviations: α, in-plane ring deformation; β, in-plane bending; γ, out-of-plane bending; δ, skeletal deformation; and ν, stretching.

^a^ Ref[35].

^b^ Ref[36].

^c^ Ref[37].

^d^ Ref[38].

^e^ Ref[39].

All three kinds of gold NPs synthesized by the fungal extract show SERS activity, but clearly, bigger NPs with complex shapes show much greater SERS activity than smaller, quasi-spherical particles.

It is well known that the optical properties of metal nanostructures are more sensitive to shape and less so to size (in contrast to semiconductor or insulator nanomaterials, which are more sensitive to size and less so to shape) [40]. However, the exact mechanism of SERS is still not completely understood. Several different theories have been developed to explain the origin of SERS, and today, it is generally agreed that the main source of enhancement is due to the amplified electromagnetic (EM) field at the surface of the metal substrate, resulting from surface Plasmon resonance of metallic nanoparticles upon excitation by the laser light used in Raman spectroscopy. Spherical NPs are in principle symmetrical, therefore presenting only one dipolar plasmon resonance while complex structures typically have multiple non-degenerated dipole modes, resulting in broad plasmon absorption spectra. The induced electronic cloud on nonspherical NPs is not distributed homogenously on the surface such that higher multipolar charge distributions are clearly induced [40]. It was shown that the EM field resulting from surface plasmon resonance of metallic nanoparticles is strongly enhanced at the corners of triangular [41-43] and decahedral [44-46] nanoparticles, with field enhancements of up to 400 times. Besides, reducing the symmetry of triangular nanoparticles resulted in higher local field-enhancements [42]. Lu et al. showed that silver nanoflowers enhanced Raman signals of Rhodamine 6G much stronger than silver nanospheres [47]. A similar effect is reported for gold nanoflowers, showing that these nanostructures enhanced Raman signals of Rhodamine B over 10 times stronger than gold nanospheres, although the latter were higher concentrated [48]. Kottmann et al. compared the local field enhancement of different nanostructures with a similar volume but different shape and showed field enhancements of 10 for elliptical, 40 for triangular, and 60 (corresponding to a Raman enhancement of 10^7^) for asymmetrical triangular nanostructures [42]. Besides, the strong field enhancements of irregular shaped nanostructures enhanced with increasing size of triangular (> 50 nm edge length) [42] and decahedral (> 60 nm edge length) [44] nanostructures. These facts can explain the greatly amplified spectrum of methylene blue on NPs of complex shapes with bigger sizes (10 to 200 nm; synthesized at 60°C and pH 3) and a relatively low SERS effect of small (3 to 12 nm), quasi-spherical NPs (synthesized at 60°C and pH 10). The stronger SERS effect of quasi-spherical NPs of 6 to 23 nm with some different shapes (synthesized at 60°C and unmodified pH), compared to the SERS effect of only quasi-spherical NPs of 3 to 12 nm, might be more likely due to the appearance of some different-shaped NPs than to insignificantly bigger sizes, based on the facts mentioned above.

Since it is well recognized that the properties of metal NPs depend greatly on their shape, size, composition, structure and crystallinity, a control over NP synthesis is required in order to fine-tune the properties of NPs with new, useful characteristics. The method discussed here demonstrates that it is possible to achieve shape tuning of NPs by using a biological system, and, as a consequence, the achievement of different SERS responses.

The strength of the EM field enhancement of irregular shaped nanostructures depends furthermore on the illumination direction [42,43] as well as the orientation and distance of the target molecule to the metal substrate [40]. Therefore, to optimize this method, more investigation is necessary, but our results are promising, indicating possible future applicability of ‘green’ synthesized NPs in various areas like biosensors and biocatalysts.

## Conclusions

In this work, we have shown for the first time that biosynthesized gold NPs display good SERS properties using methylene blue as a target molecule, presenting promising results and showing that these green synthesized gold NPs might have potential applications in sensor technology. Furthermore, results clearly indicate that size and shape of the NPs produced can easily be tuned by changing the environmental conditions such as temperature and pH value.

## Supporting Information

Figure S1 **Statistical analysis.** ANOVA of gold nanoparticles synthesized under different pH values and at different time points. A: Average sizes of nanoparticles at different time points with respect to different pH values. B: Tukey’s HSD *post* *hoc* test analysis with average nanoparticle size information. Vertical bars denote 0.95 confidence intervals. Significant differences are marked in red.
(TIF)

Figure S2 **NP Stability.** Size distribution histograms of gold NPs synthesized at 60°C and pH 3 (A), pH 5.5 (B), and pH 10 (C) after more than 10 months storage at room temperature.
(TIF)
