# Supplementary figures and images for: SERS Properties of Different Sized and Shaped Gold Nanoparticles Biosynthesized under Different Environmental Conditions by Neurospora crassa Extract
Source: PLoS One. 2013 Oct 9;8(10):e77486. doi: 10.1371/journal.pone.0077486 (PMC3793955; doi:10.1371/journal.pone.0077486)

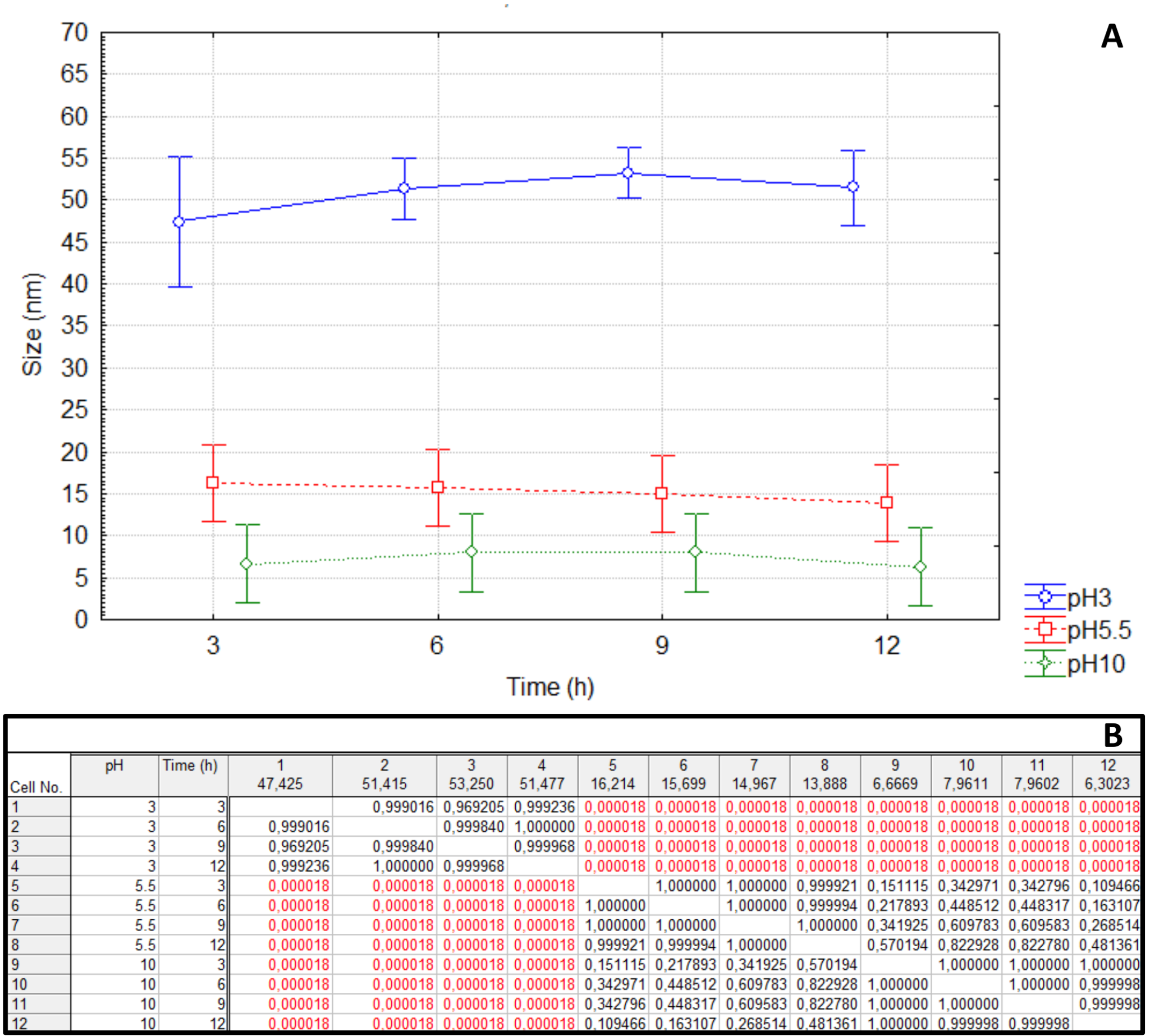

Supplement: Figure S1 — Statistical analysis. ANOVA of gold nanoparticles synthesized under different pH values and at different time points. A: Average sizes of nanoparticles at different time points with respect to different pH values. B: Tukey’s HSD post hoc test analysis with average nanoparticle size information. Vertical bars denote 0.95 confidence intervals. Significant differences are marked in red. (TIF) [file pone.0077486.s001.tif]
